# Micro(nano)plastics: invisible compounds with a visible impact

**DOI:** 10.12688/f1000research.142212.2

**Published:** 2024-04-19

**Authors:** Prabhakar Sharma, Prateek Sharma

**Affiliations:** 1 Department of Agricultural Engineering and Technology, School of Engineering, Nagaland University, Dimapur, Nagaland, 797112, India; 2 Environmental Science, Central University of Jharkhand, Ranchi, Jharkhand, 835222, India

**Keywords:** Micro(nano)plastics; groundwater; separation method; remediation

## Abstract

The plastic related research has been an epicentre in recent times. The presence and spread of micro (nano) plastics (MNPs) are well-known in the terrestrial and aquatic environment. However, the focus on the fate and remediation of MNP in soil and groundwater is limited. The fate and bioaccumulation of ingested MNPs remain unknown within the digestive tract of animals. There is also a significant knowledge gap in understanding the ubiquitous organic environmental pollutants with MNPs in biological systems. Reducing plastic consumption, improving waste management practices, and developing environmentally friendly alternatives are some of the key steps needed to address MNP pollution. For better handling and to protect the environment from these invisible substances, policymakers and researchers urgently need to monitor and map MNP contamination in soil and groundwater.

## Introduction

**Figure 1.**
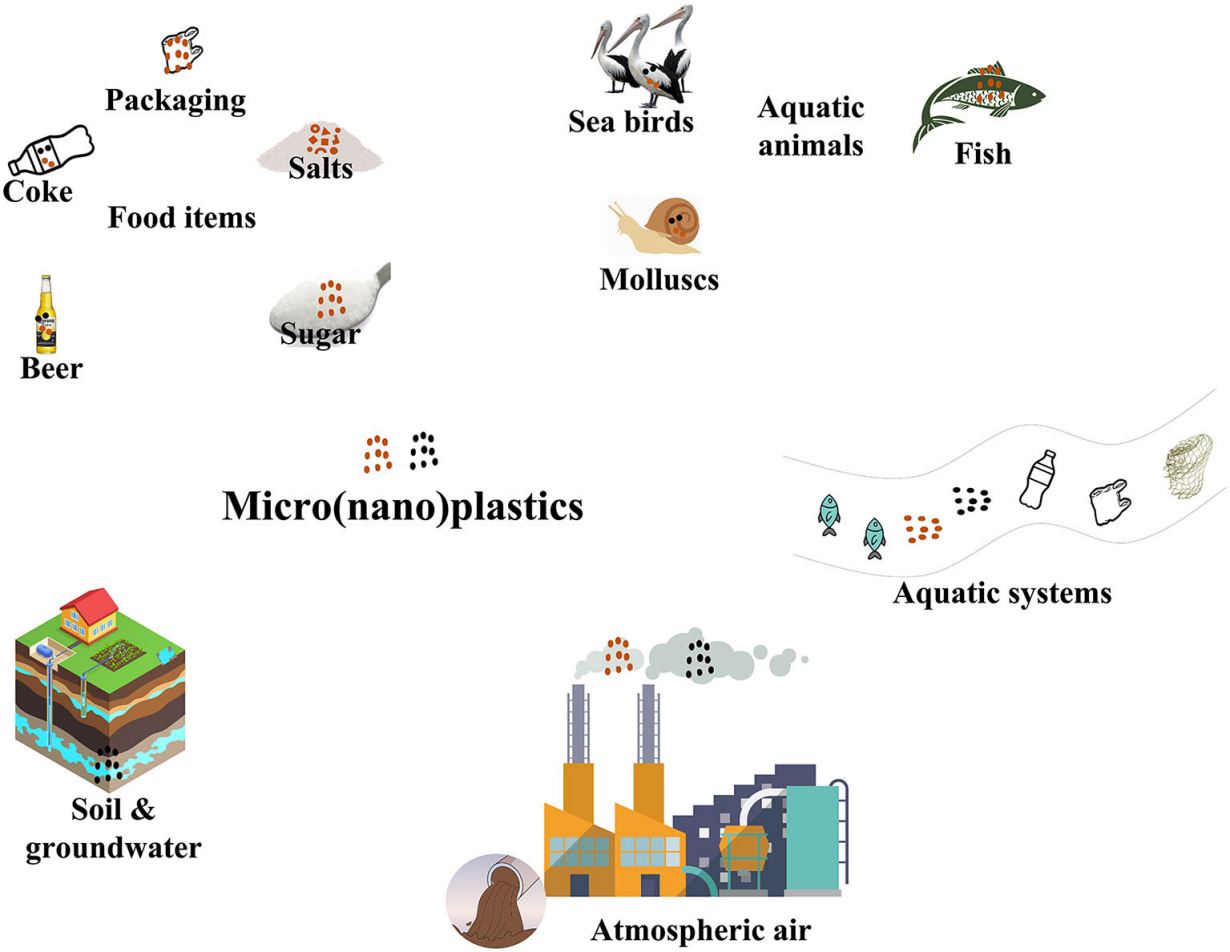
The interference of MNPs in different environments.

**Figure 2.**
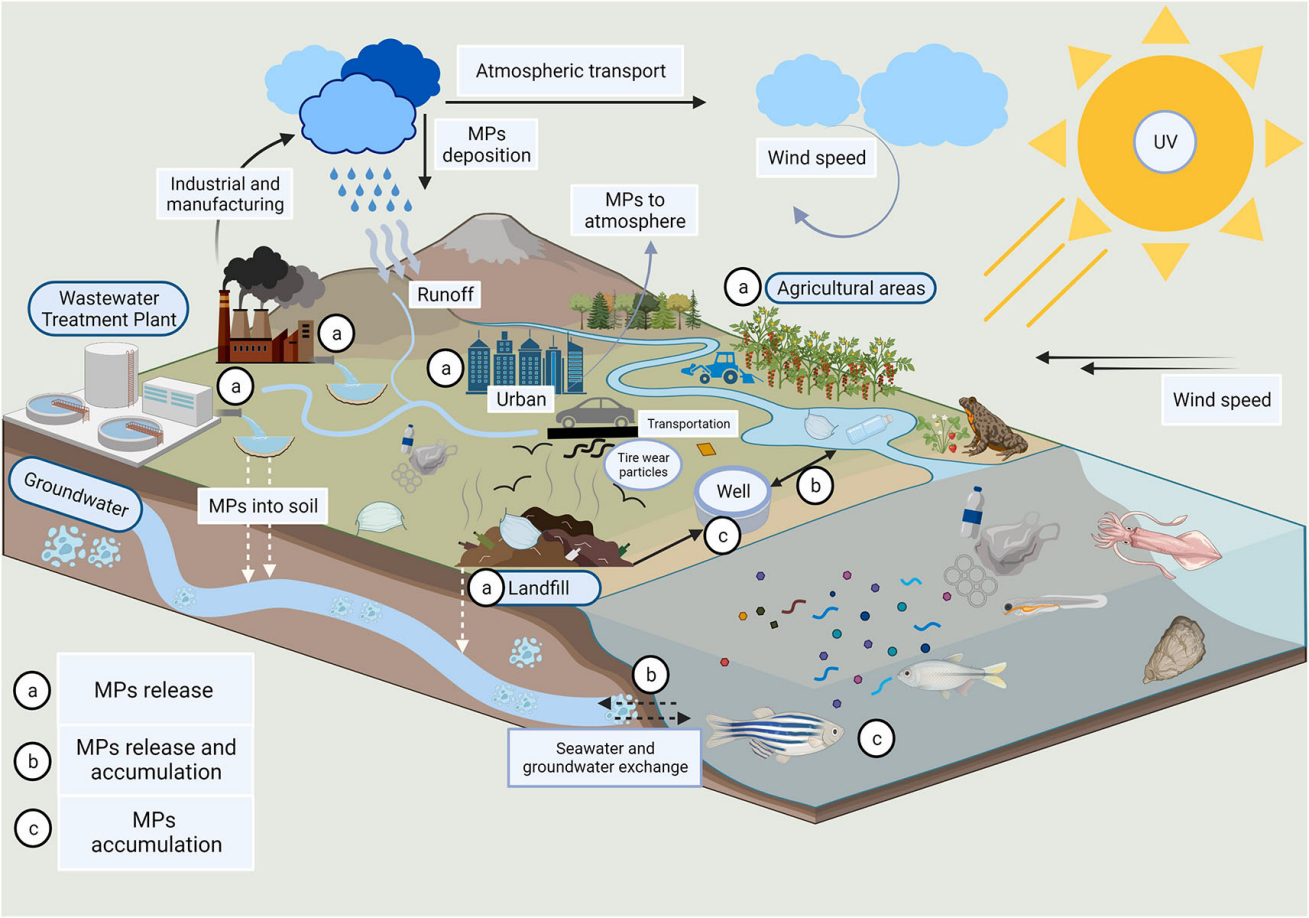
The presence of invisible plastic compounds in groundwater (adapted from Sangkham et al.; Elsevier License Number: 5653250836205). ^28^

The invention of plastic was a terrific innovation; now, it has penetrated everywhere, and the estimated plastic production reached 367 million tonnes in 2020 globally. ^1^ Plastic is a synthetic polymer made from crude oil and natural gas. The dependence of the public on plastic merchandise has inevitably led to a prompt rise in plastic manufacturing volume. Plastic-related research is increasingly in focus in recent times and more volume of studies are being pursued. ^2^^,^^3^ Due to the presence of different forms of plastics, the waterbodies (ocean, river, lake, wetland, etc), the atmosphere, the terrestrial environment, and even the groundwater are being polluted by microplastics (<5 mm) and nanoplastics (<100 nm) ( Figure 1). ^4^ The large plastics products are fragmented into smaller pieces and get converted into MNPs through various processes. ^5^^,^^6^ At the same time, the hydrocarbon chains in plastic compounds make them difficult for plastic wastes to decompose in the environment. MNPs are reaching either directly into the environment or after the degradation of plastic debris. Although the MNPs have different sizes (1 nm to 5 mm), various shapes (fibre, film, foam, etc.), and unique chemical structures (polyethene, polypropylene, polyethene terephthalate, etc.), they are generally invisible in nature. MNPs not only pose direct threats to marine life through ingestion but also serve as carriers for toxic chemicals, further amplifying their ecological impact. Understanding their distribution, behavior, and long-term effects is crucial for developing effective strategies to mitigate their pervasive presence in the environment. At the same time, MNPs interact with the neighbouring compounds and microorganisms, which is crucial for animal, human, and plant health; *i.e.*, these invisible compounds comfortably react/interact with the surrounding environment, including soil, organic and inorganic compounds, microorganisms, heavy metals, and plants. ^7^ As a result, MNPs-related research has been getting significant attention in recent years, both in societies and in scientific communities, due to their potential impacts on ecological systems and human health. ^8^^,^^9^ Groundwater contamination by plastic microfibers is a pressing concern in environmental science ( Figure 2). ^10^ These tiny, nearly invisible plastic fibres shed from clothing, textiles, and other sources can infiltrate the soil and eventually reach groundwater reserves. Once there, they pose a risk of contamination, potentially affecting both aquatic ecosystems and human drinking water sources. ^10^ Understanding and mitigating this hidden threat is essential for safeguarding our precious groundwater resources and the overall health of our environment. However, the current research on interactions of these invisible MNPs is in its early stages and data are limited in the important connected sectors. The objective of this article is to highlight the perspectives of current characterization methodologies, health impact, important source pathways, and available remediation options for the invisible MNPs present in the environment.

## MNPs Separation and Quantification Methods

**Figure 3.**
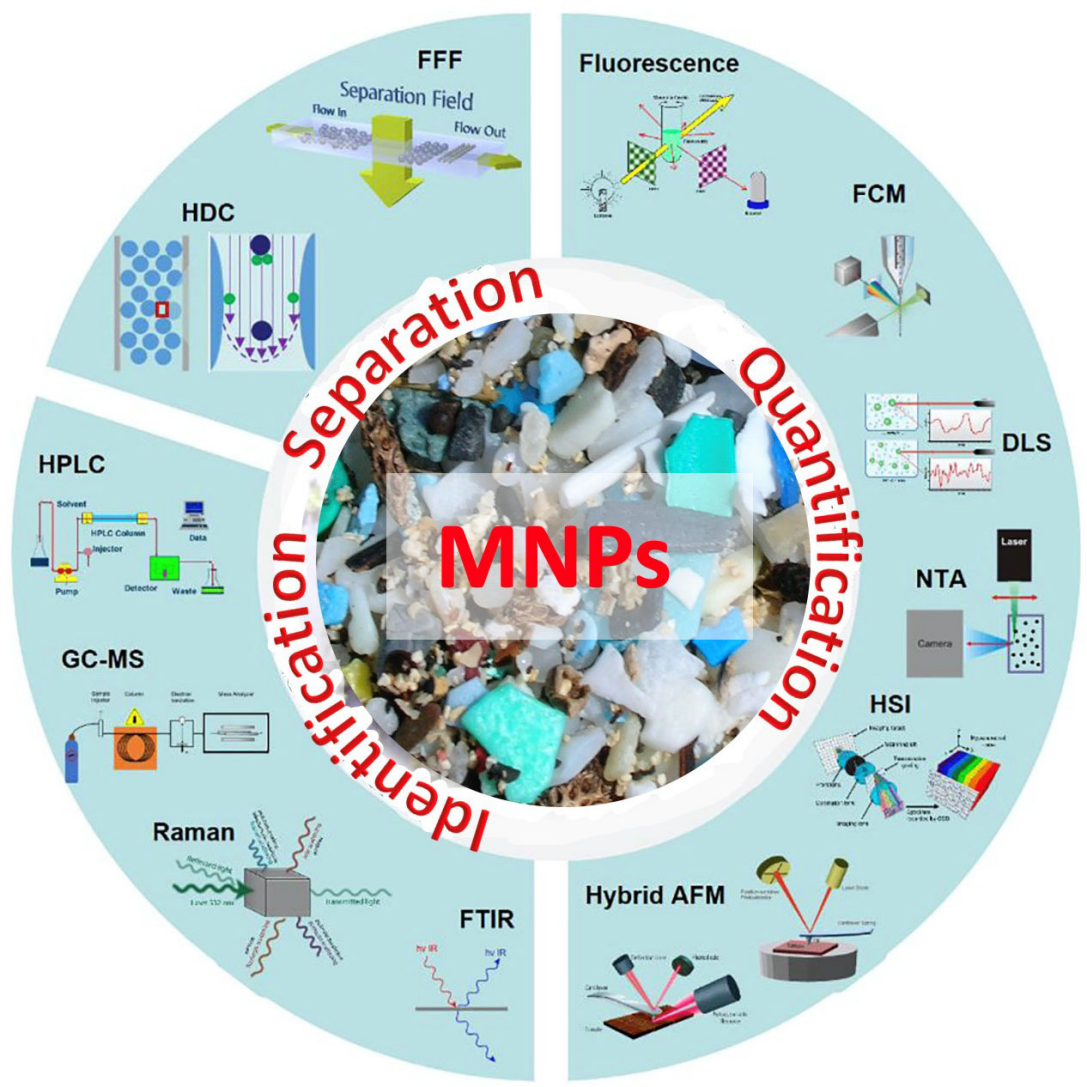
The separation, quantification, and identification methodology of MNPs.

The quantitation and analysis of MNPs are essential for accurately assessing their existence and intensity in different environmental samples. ^11^ A number of techniques are employed for quantification depending on the type of MNPs sample and the size range of MNPs being investigated such as density separation and filtration, ^12^ microscopic and spectroscopic techniques, ^11^ chromatographic approach, ^13^ nanoparticle tracking analysis (NTA), ^14^ quantitative polymerase-chain-reaction (qPCR) approach ^15^ ( Figure 3).

Density separation and filtration are common tools to isolate and concentrate MNPs from soil or water samples. It is employed to segregate MNPs from the collected soil or water samples for further characterization. Optical microscopy is a traditional method of visualization of MNPs. In this method of direct visualization, the segregated MNP samples are collected and prepared on glass slides, and they are manually counted and quantified. Although this method can provide valuable information about the presence of MNPs, it may not be suitable for quantifying relatively small particles or dealing with large sample sizes. However, infrared or Raman spectroscopy is being used for identifying and quantifying MNPs based on unique molecular vibrations of plastic compounds to better understand the polymeric types of these invisible plastic compounds present in the environmental sample. ^11^

More recently, a combination of microscopy and infrared spectroscopy has been employed to identify and quantify MNPs in individual samples. ^11^ They provide spatial distribution and allow for a more detailed characterization of MNPs in complex soil or water samples. The pyrolysis-gas chromatography-mass spectrometry is also used to identify and quantify MNPs based on the polymer’s chemical breakdown (pyrolysis process). ^13^ It conveniently provides information about the types and concentrations of plastics in the soil or water sample.

In addition, nanoparticle tracking analysis (NTA) has also been considered an optical technique for tracking individual MNP particles based on their Brownian motion. ^14^ It provides information on particle size distribution and concentration as well. More recently, quantitative polymerase chain reaction (qPCR), a technique within molecular biology based on MNP ingested by microorganisms or organisms, has also been practised. ^15^ All of these techniques for quantifying and analysing MNPs have benefits and drawbacks, but the choice of technique mostly depends on the kind of sample, the goals of the research, and the resources at hand. In order to do more thorough analyses of these invisible plastic compounds in environmental samples, a combination of various approaches is frequently used. To guarantee the accuracy and comparability of MNP data across the globe, these methodologies must be standardised.

## Impact of MNPs on the Environment

The potential impact of MNPs on the environment and human health is a noble area of research. ^16^ The extent of the health impacts of MNPs is not yet fully understood, several potential pathways of exposure and adverse health outcomes have been recognised. It is important to note that the current scientific understanding of MNP toxicity on human health is still in its early stages, ^16^ and much more research is needed to fully understand the extent of the risk. The presence of these invisible plastic compounds in the human body and their potential health effects have prompted calls for more comprehensive studies, improved waste management, and the development of sustainable alternatives to reduce plastic pollution and potential human exposure.

### Direct impact on human and animal health

Micro (nano) plastics can directly impact the aquatic animal and the human food chain. More recently, they occurred in human blood and other organs with a massive implication for human health in causing cancer or other diseases. ^17^^,^^18^ Generally, MNPs accumulation in marine organisms at the lower trophic levels poses potential health risks as they are being consumed by the higher trophic levels and by humans mostly relying on seafood. The intake of MNPs causes oxidative stress, inflammatory reactions, fitness disturbances, and endocrine disorders in humans. ^19^ As they penetrate the body cells, they create cytotoxicity, DNA oxidative damage, genotoxicity, reactive oxygen species (ROS) increase, and increased stream in genes. Micro (nano) plastics induce oxidative stress in the body by releasing chemicals and ROS which leads to cancer development by causing mechanical and genetic damage. ^20^ The accumulation of MNPs in the digestive tract of animals is a growing environmental concern. When animals mistakenly ingest plastic materials like bags, bottles, or other MNP compounds, they can become lodged in their stomach or intestine, causing a range of serious health issues. These foreign objects not only obstruct the digestive process but can also leach harmful chemicals into the animal's body. In many cases, this plastic ingestion can be fatal, posing a significant threat to wildlife populations and highlighting the urgent need for global efforts to reduce plastic pollution and protect our ecosystems. These invisible plastic compounds infiltrate directly into the intestinal mucosa while mingling with other intestinal content and causing inflammations and reduction in intestinal mucus secretion, intestinal barrier dysfunction, increased gut mucosa permeability, and imbalance of gut microbiota. ^21^ Micro (nano) plastics also enter the gastrointestinal tract via the epithelial route then they cause physical damage in the gastrointestinal tract of humans. In animal studies, ingested MNPs could cause inflammation, tissue damage, and alterations in gut microbiota, which could impact overall gut health. ^22^ Exposure to MNPs may trigger immune responses in the body, leading to inflammation and potential immune system dysregulation. Long-term immune system perturbations could also increase susceptibility to infections and other health issues. Some studies suggest that MNPs may have endocrine-disrupting properties in the affected animals, interfering with hormone signalling in the body. ^18^ This interference potentially leads to reproductive and developmental issues for humans and animals. The existing literature suggests that MNPs can accumulate in various organs and tissues, raising concerns about potential long-term health effects related to chronic exposure. ^12^ The ingestion of these invisible plastic compounds by a human lead to cytotoxicity and similar colonic cancer, as stool samples are a potent source of identification of MNPs. ^23^

### Impact due to associated pollutants

Micro (nano) plastics have been found in a wide range of food items, including seafood, drinking water, and even salt ( Figure 1). ^24^ These items may pose health risks due to their potential to release not only MNPs but also the associated chemical additives and adsorbed toxic substances. As the heavy metals or organic pollutants increase in the environment, the large specific surface area and porous structure of MNPs make them easier to adsorb and enrich other environmental pollutants. These MNPs-contaminant complexes may lead to ecological disturbances. ^7^ Morphologically, these MNPs may act together with other stressors (environmental contaminants, drought stress, or salinity) to produce a combined effect; however, it is unknown whether this combined effect is the sum of the individual effects or a synergistic or antagonistic effect, *i.e.*, the effects of MNPs-contaminant complexes on ecosystem structures and functions or their response thresholds. ^25^

The synergistic effect occurs when the combination of MNPs with other stressors or pollutants lead to a more substantial impact than the sum of their individual effects. This process may enhance the toxic effects of MNP to animals or humans by reducing the biodegradation rate after entering their bodies. ^26^ Micro (nano) plastics can adsorb and accumulate chemical pollutants from the environment, such as pesticides, heavy metals, and persistent organic pollutants. ^25^ In this case, MNPs act as carriers by facilitating the transfer of harmful chemicals into the food web or to the organisms. The combined toxicity of MNPs and adsorbed chemicals can result in more severe health effects on aquatic organisms and potentially humans when consuming contaminated seafood. ^26^ In some instances, the presence of MNPs may encourage the development of toxic algal species, resulting in harmful algal blooms that have a negative impact on the water quality and marine life. ^27^ Micro (nano) plastics can physically injure organisms by abrading their surfaces or obstructing their gastrointestinal tracts. ^28^ The detrimental effect on an organism's health and survival is exacerbated when combined with other environmental stresses, such as temperature changes or decreased food supply. Antagonistic effects occur when the presence of MNPs transforms the behaviour or uptake of other pollutants, potentially reducing their toxicity. ^29^ While some studies have suggested that MNPs might reduce the bioavailability of certain contaminants, leading to lower uptake by organisms, the overall antagonistic impact is not yet fully understood. The presence of MNPs might reduce the bioavailability of harmful chemicals by creating a physical barrier that limits the direct contact between contaminants and organisms. This could lead to reduced uptake of toxins by organisms, potentially mitigating some of the toxic effects as well.

## Source Pathways of MNPs

Although the current literature had properly covered the source, pathways, quantification, and even interaction of these invisible MNPs. The MNPs originate from a multitude of sources, including the fragmentation of larger plastic debris, the shedding of microfibers from synthetic textiles during laundering, the abrasion of tires on roads, the release of microbeads from personal care products, and the breakdown of industrial plastic pellets (nurdles). These MNPs can enter the environment through various pathways, including direct deposition, atmospheric deposition, surface runoff, and wastewater discharge. So it is crucial to understand the sources and pathways of MNPs of the contaminated sites to develop effective mitigation strategies and prevent further pollution. ^30^ For example, the release of fibre from textiles, the presence of microbeads in cosmetic products, and the degradation of plastic debris could be potential sources of MNPs. ^31^ Most of this research emphasizes the amount and degradation of plastics, recognition and quantification of MNPs in the environment and in the microorganisms, toxicological effects on single species, and combined effects with others pollutantss. The presence of MNPs in the environment poses numerous ecological and human health risks. MNPs can be ingested by a wide range of organisms, leading to physical harm, internal blockages, and potential transfer through the food chain. MNPs can adsorb and transport toxic chemicals, including persistent organic pollutants and heavy metals, thereby magnifying their toxicity and bioavailability. Additionally, MNPs have been linked to adverse effects on reproductive health, immune function, and developmental processes in aquatic organisms. However, there are still some critical issues that urgently need to be addressed. Such as the impact of these invisible plastic compounds on the structural stability of ecosystems or their remediation, especially from the soil and groundwater, is limited. ^32^ The land-use pattern and soil types play a critical role in the infiltration of MNPs into subsurface systems and groundwater. ^33^

The leaching of MNPs can happen predominantly from agricultural soils or from urban infrastructure wastes. The improper disposal of plastic waste, such as plastic bags, bottles, and packaging materials, is a significant source of MNPs in the environment. Plastics that end up as litter on land can be transported by wind, rain, and water runoff into aquatic bodies. Generally, larger plastic items, such as bottles and bags, break down into MNPs through processes like photodegradation, mechanical weathering, and chemical degradation. They are called secondary MNPs. However, the primary MNPs have resulted from synthetic textiles, like polyester and nylon, shed microfibers during laundering, wear down of vehicle tires over time, industrial wastes containing plastic pellets (known as nurdles), as well as microbeads from personal care products. The biosolids treated from sewage sludge applied on agricultural fields are also a critical source of these invisible plastic compounds in soil and groundwater. ^34^

Although a large volume of literature has focused on the presence of invisible MNPs in marine and freshwater systems, some of the recent studies have started focusing on soil, groundwater, and remote locations. ^32^^,^^33^ Surface water bodies such as rivers, lakes, and oceans are often the first recipients of MNPs from various sources, including urban runoff, industrial discharges, and plastic debris from land. All of these investigations have shown that MNP pollution of surface waterways is widespread, with significant concentrations occurring around urban areas or plastic manufacturing facilities.

The studies on MNPs in soil are relatively new and not as abundant as those of surface water, soil contamination by MNPs also occurs through various pathways, including the direct application of plastic mulches, sewage sludge, and atmospheric deposition. A common methodology of MNPs separation and characterization is being adopted for soil as well. While MNP concentrations in soil may not be as high as in surface waters, they can still pose a risk to terrestrial ecosystems and potentially enter the food chain through plant uptake. ^32^ Further, groundwater contamination can occur through the percolation of MNPs from surface soils, landfills, and septic systems which raises concern about their potential impacts on drinking water quality and groundwater-dependent ecosystems. ^35^^,^^36^ Due to the challenges of accessing and sampling groundwater, research in this area has been relatively challenging. ^32^^,^^37^ To investigate MNP content in groundwater, researchers have used well water sampling and then proceeded with common filtration, separation, and microscopic techniques. ^33^

In short, studies on MNPs in surface water are more extensive and established compared to those in soil and groundwater but the potential source and the presence of MNPs in all three compartments have been confirmed, and each environment poses unique challenges for research and monitoring. To create efficient mitigation methods and protect both human health and the environment, it is crucial to keep researching the source pathways, fate, and potential effects of MNPs on these environmental compartments.

## Role of MNPs on Ecosystem Functions

Plastics are polymers based on chains of carbon atoms and carbon as the main component. Micro (nano) plastic releases in the dissolved form as organic carbon or as a greenhouse gas (such as CO _2_) while degrading in the ecosystem. So, MNPs are also closely associated with the current climate crisis because they are directly or indirectly involved in greenhouse gas emissions from their production to disposal stages. ^38^

Micro (nano) plastics have a significant effect on ecosystem functions, leading to ecological disruptions and alterations in natural processes. ^39^ These effects can impact both aquatic and terrestrial ecosystems. They alter the nutrient cycling of the ecosystem when ingested by organisms. Micro (nano) plastics have an impact on biodiversity, community structure, and the distribution of species within ecosystems. Other species that rely on them for food or ecological interactions are affected in a cascade manner by this. The accumulation of MNPs in sediments, soils, and water bodies modifies the habitat, bioaccumulates the hazardous substances around it, changes how different animals feed and reproduce, and lowers the ecosystem's ability to withstand environmental stressors. ^39^ All these have long-term consequences for population dynamics and ecosystem stability. Organisms exposed to MNPs may be more vulnerable to other ecological pressures, such as changes in temperature, salinity, or pollution, leading to greater susceptibility to diseases and overall ecosystem instability. Micro (nano) plastics also affect the delivery of ecosystem services to human populations and decrease agricultural productivity and fisheries yield, and affect the carbon sequestration capacity of ecosystems. ^40^ Micro (nano) plastics influence the carbon burial rates in marine sediments and ultimately affect the carbon sequestration capacity of ecosystems and alter the global carbon cycle. The intentional and unintentional input of these invisible plastic compounds into the environment may have a latent impact on global carbon stocks. So, a strong commitment is required to find a replacement for plastic products.

## Remediation Strategies for MNPs

The remediation of MNPs is a challenging task due to their widespread distribution, small size, persistence in terrestrial and aquatic ecosystems and their consequent impacts on natural systems. ^41^ The removal of MNPs from the contaminated ecosystem is necessary for a benign environment. Two strategies have been used in practice to systematically manage or minimise the pollution caused by MNPs: the first is to block the natural ecosystem's access points, and the second is to remove MNPs *in situ* from the already contaminated bodies. However, researchers are further exploring various other strategies to address the issue. For example, the upgradation of existing wastewater treatment technologies using granular activated carbon filtration, membrane filtration, and ozonation has a significant impact on MNPs removal. At the same time, stormwater management and beach clean-up initiatives have a promising outcome for preventing the generation of secondary MNPs. Some microorganisms and specialized filter-feeding organisms have also shown the ability to consume and break down MNPs. ^41^ The development and use of biodegradable and environmentally friendly plastics can help reduce the accumulation of MNPs in the environment. Increasing public awareness about MNP pollution and educating people about the sources and impacts of MNPs can help reduce their release into the environment.

The reduction of MNPs using biochar is another emerging area of research and holds promise as a potential solution to tackle MNP pollution. ^42^ According to Wang *et al.,* ^43^ biochar is an effective and affordable investment in the removal of MNPs. They observed that sand filters mediated with biochar enhanced the removal efficiency of MNPs. The porous nature of biochar offers a lot of binding sites and a lot of surface area, making it a good adsorbent for MNPs. ^42^ Biochar attracts and traps MNP after being added to contaminated settings, such as soil or water, by limiting its mobility and availability to living things. To lessen the movement of these invisible plastic compounds from sediments to water columns during erosion events or tidal action, biochar can be used as a sediment amendment in coastal locations. It was observed that biochar can help MNPs degrade more quickly by enhancing the vital microbial communities. ^42^

## Innovative Solutions and Ongoing Efforts for Mitigation of MNPs

In the ongoing battle to mitigate the detrimental effects of MNP on our environment, innovative solutions and concerted efforts have emerged as critical strategies. The preventive measures include reducing plastic consumption, improving waste management practices, implementing bans on microplastics in consumer products, and promoting the use of biodegradable alternatives. The remediation strategies encompass techniques such as filtration, sediment dredging, and the development of innovative nanomaterial-based sorbents for capturing MNPs from water and soil environments. Furthermore, enhanced monitoring and surveillance programs are essential for assessing the distribution, abundance, and ecological impacts of MNPs, thereby guiding targeted interventions and policy decisions. The cutting-edge wastewater treatment technologies are being developed to capture and filter out these minuscule plastic particles before they reach our water bodies, significantly reducing their input. ^35^ Biodegradable plastics are also under development to curtail the persistence of MNPs in the environment, though challenges remain in their widespread adoption. Nanotechnology offers promise with materials like magnetic nanoparticles that can attract and remove microplastics from water sources. Furthermore, regulatory bodies are increasingly implementing bans and restrictions on single-use plastics, while citizen science initiatives and educational campaigns are raising awareness and contributing to data collection efforts. Innovations in cleanup technologies for oceans and waterways, as well as a shift towards a circular economy, underscore the diverse range of approaches being pursued to combat the MNP crisis. These collective endeavours aim to safeguard ecosystems and human health from the pervasive impact of these invisible plastic compounds.

## Strategies and Future Recommendations

As plastic-related research is an epicentre from the past decade, this paper emphasizes the technological advancements in MNP research and development with their health implications, types, sources, and environmental implications.

• Although the presence and spread of MNPs are well-known in the terrestrial and aquatic environment, the focus on the fate of MNP in groundwater and their remediation process is scarce. More studies are required to understand the properties, concentrations, and types of MNPs in soil and groundwater in order to assess the public health risks. Therefore, it is urgently required to delineate MNP pollution by the policymaker and researcher for their monitoring, assessment, and handling to safeguard the environment from these invisible compounds.
• A local-to-global scale mapping of MNP pollution is required in the future by the policymakers, and society must act on their reduction at the local to global level from daily uses.
• To address the impacts of MNPs on the environment and human health, it is crucial to continue research, implement proper waste management practices, reduce plastic use and promote sustainable alternatives, and develop innovative remediation technologies. A proper understanding of the interactions between MNPs and ecosystems is essential for developing effective conservation and mitigation strategies to safeguard ecosystem health and the services they provide.
• Addressing the impact of MNPs on ecosystem functions requires a holistic approach, including reducing plastic pollution at its source, implementing effective waste management, and promoting sustainable practices to minimize plastic usage.
• Even today, there is still a lack of effective technical means to accurately quantify these invisible MNPs in the environment, which hinders their assessment of toxicological effects and ecological risks. So, the standardization of plastics pollution and its remediation from the environment is required across the globe. Overall, the adverse environmental impact of MNPs and their standard treatment strategies should urgently be practised by different stakeholders across the globe.

## Declarations

## Authors’ contributions

Prabhakar Sharma and Prateek Sharma conceived and planned the study, wrote the main manuscript text, and reviewed the manuscript. Prabhakar Sharma prepared the figure.

## Ethical approval and consent to participate

Not applicable.

## Consent for publication

Not applicable.

## Data Availability

There are no data associated with this article. All the data used in this paper has been provided.

## References

[1] Plastics Europe: Plastics — the Facts 2021: An analysis of European plastics production, demand and waste data. 2021.

[2] Bhat M Eraslan F Gaga E : Scientometric analysis of microplastics across the globe.2023;1–13. 10.1002/9781119879534.ch1

[3] Bhat MA Gedik K Gaga EO : Atmospheric micro (nano) plastics: future growing concerns for human health. Air Qual. Atmos. Health. 2023;6(2):233–262. 10.1007/s11869-022-01272-2 PMC9574822 36276170

[4] Rochman CM : Microplastics research—from sink to source. *Science.* 2018;360:28–29. 10.1126/science.aar7734 29622640

[5] Bhat M : Unveiling the overlooked threat: Macroplastic pollution in indoor markets in an urban city. Case Stud. Chem. Environ. Eng. 2024;9. 10.1016/j.cscee.2023.100558

[6] Bhat M Gedik K Gaga E : A preliminary study on the natural aging behavior of microplastics in indoor and outdoor environments. Int. J. Environ. Sci. Technol. 2024;21(2):1923–1936. 10.1007/s13762-023-05319-4

[7] Kumar R Ivy N Bhattacharya S : Coupled effects of microplastics and heavy metals on plants: uptake, bioaccumulation, and environmental health perspectives. *Sci. Total Environ.* 2022;836:155619. 10.1016/j.scitotenv.2022.155619 35508241

[8] Thacharodi A Hassan S Meenatchi R : Mitigating microplastic pollution: A critical review on the effects, remediation, and utilization strategies of microplastics. J. Environ. Manage. 2024;351: 119988. 10.1016/j.jenvman.2023.119988 38181686

[9] Thacharodi A Meenatchi R Hassan S : Microplastics in the environment: A critical overview on its fate, toxicity, implications, management, and bioremediation strategies. J. Environ. Manage. 2024;349. 10.1016/j.jenvman.2023.119433 39492398

[10] Re V : Shedding light on the invisible: addressing the potential for groundwater contamination by plastic microfibers. *Hydrogeol. J.* 2019;27:2719–2727. 10.1007/s10040-019-01998-x

[11] Raj D Maity SK : Critical assessment of approach towards estimation of microplastics in environmental matrices. *Land Degrad. Dev.* 2023;34(10):2735–2749. 10.1002/ldr.4665

[12] Han X Lu X Vogt RD : An optimized density-based approach for extracting microplastics from soil and sediment samples. *Environ. Pollut.* 2019;254(A):113009. 10.1016/j.envpol.2019.113009 31419661

[13] Xu Y Qu Q Jiao M : Identification and quantification of nanoplastics in surface water and groundwater by pyrolysis gas chromatography-mass spectrometry. *Environ. Sci. Technol.* 2022;56(8):4988–4997. 10.1021/acs.est.1c07377 35373559

[14] Peters RJW Relou E Sijtsma ELE : Evaluation of nanoparticle tracking analysis (NTA) for the measurement of nanoplastics in drinking water. *Int. J. Food Contam.* 2023. 10.21203/rs.3.rs-1809144/v1

[15] Pizzoferrato R Li Y Nicolai E : Quantitative detection of microplastics in water through fluorescence signal analysis. *Photonics.* 2023;10(5):508. 10.3390/photonics10050508

[16] Vethaak AD Legler J : Microplastics and human health. *Science.* 2021;371:672–674. 10.1126/science.abe5041 33574197

[17] Leslie HA Velzen MJM van Brandsma SH : Discovery and quantification of plastic particle pollution in human blood. *Environ. Int.* 2021;163:107199. 10.1016/j.envint.2022.107199 35367073

[18] Kumar R Manna C Padha S : Micro (nano) plastics pollution and human health: How plastics can induce carcinogenesis to humans? *Chemosphere.* 2022;298:134267. 10.1016/j.chemosphere.2022.134267 35301996

[19] Wang W Gao H Jin S : The ecotoxicological effects of microplastics on aquatic food web, from primary producer to human: A review Ecotoxicology and environmental Safety. 2019;173:110–117. 10.1016/j.ecoenv.2019.01.113 30771654

[20] Schirinzi GF Pérez-Pomeda I Sanchís J : Cytotoxic effects of commonly used nanomaterials and microplastics on cerebral and epithelial human cells. *Environ. Res.* 2017;159:579–587. 10.1016/j.envres.2017.08.043 28898803

[21] Rahman A Sarkar A Yadav OP : Potential human health risks due to environmental exposure to microplastics and knowledge gaps: a scoping review. *Sci. Total Environ.* 2020;757:143872. 10.1016/j.scitotenv.2020.143872 33310568

[22] Gruber ES Stadlbauer V Pichler V : To waste or not to waste: Questioning potential health risks of micro- and nanoplastics with a focus on their ingestion and potential carcinogenicity. *Expo Health.* 2023;15:33–51. 10.1007/s12403-022-00470-8 36873245 PMC9971145

[23] Ibrahim YS Tuan Anuar S Azmi AA : Detection of microplastics in human colectomy specimens. *JGH Open.* 2021;5:116–121. 10.1002/jgh3.12457 33490620 PMC7812470

[24] Altunisik A : Prevalence of microplastics in commercially sold soft drinks and human risk assessment. *J. Environ. Manag.* 2023;336:117720. 10.1016/j.jenvman.2023.117720 36907066

[25] Budhiraja V Urh A Horvat P : Synergistic adsorption of organic pollutants on weathered polyethylene microplastics. *Polymers.* 2022;14(13):2674. 10.3390/polym14132674 35808719 PMC9269090

[26] Martin J Santos JL Aparicio I : Microplastics and associated emerging contaminants in the environment: Analysis, sorption mechanisms and effects of co-exposure Trends in Environmental Analytical Chemistry. 2022;35:e00170.

[27] Pasqualini V Garrido M Cecchi P : Harmful algae and pathogens on plastics in three mediterranean coastal lagoons. *Heliyon.* 2023;9(3):e13654. 10.1016/j.heliyon.2023.e13654 36895393 PMC9988496

[28] Rodrigues ACB Jesus GP de Waked D : Scientific evidence about the risks of micro and nanoplastics (MNPLs) to human health and their exposure routes through the environment. *Toxics.* 2022;10(6):308. 10.3390/toxics10060308 35736916 PMC9228263

[29] Kaur H Rawat D Poria P : Ecotoxic effects of microplastics and contaminated microplastics - Emerging evidence and perspective. *Sci. Total Environ.* 2022;841:156593. 10.1016/j.scitotenv.2022.156593 35690218

[30] Abbasi S : Routes of human exposure to micro (nano)plastics. *Curr. Opin. Toxicol.* 2021;27:41–46. 10.1016/j.cotox.2021.08.004

[31] Bhat M Eraslan F Gedik K : Impact of textile product emissions: toxicological considerations in assessing indoor air quality and human health.springer.2021. 10.1007/978-3-030-76073-1_27

[32] Li S Ding F Flury M : Macro- and microplastic accumulation in soil after 32 years of plastic film mulching. *Environ. Pollut.* 2022;300:118945. 10.1016/j.envpol.2022.118945 35122919

[33] Sangkham S Islam MA Adhikari S : Evidence of microplastic contamination in groundwater and human health risk assessment perspectives: A review. *Groundw. Sustain. Dev.* 2023;23:100981. 10.1016/j.gsd.2023.100981

[34] Manikanda BK Natesan U Vaikunth R : Spatial distribution of microplastic concentration around landfill sites and its potential risk on groundwater. *Chemosphere.* 2021;277:130263. 10.1016/j.chemosphere.2021.130263 33770695

[35] Chia RW Lee JY Kim H : Microplastic pollution in soil and groundwater: a review. *Environ. Chem. Lett.* 2021;19:4211–4224. 10.1007/s10311-021-01297-6

[36] Singh S Bhagwat A : Microplastics: a potential threat to groundwater resources. *Groundw. Sustain. Dev.* 2022;19:100852. 10.1016/j.gsd.2022.100852

[37] Khant N Kim H : Review of current issues and management strategies of microplastics in groundwater environments. *Water.* 2022;14(7):1020. 10.3390/w14071020

[38] Bergmann M Collard F Fabes J : Plastic pollution in the Arctic. *Nat. Rev. Earth Environ.* 2022;3(5):323–337. 10.1038/s43017-022-00279-8

[39] Green DS Boots B O’Connor NE : Microplastics affect the ecological functioning of an important biogenic habitat. *Environ. Sci. Technol.* 2017;51(1):68–77. 10.1021/acs.est.6b04496 27936642

[40] Shafea L Yup J Beriot N : Microplastics in agroecosystems: A review of effects on soil biota and key soil functions. *J. Plant Nutr. Soil. Sci.* 2023;186(1):5–22. 10.1002/jpln.202200136

[41] Mishra SR Ahmaruzzaman M : Microplastics: identification, toxicity and their remediation from aqueous streams. *Sep. Purif. Rev.* 2022;52:283–304. 10.1080/15422119.2022.2096071

[42] Kumar R Verma A Rakib MRJ : Adsorptive behavior of micro (nano) plastics through biochar: Co-existence, consequences, and challenges in contaminated ecosystems. *Sci. Total Environ.* 2023;856:159097. 10.1016/j.scitotenv.2022.159097 36179840

[43] Wang Z Sedighi M Lea-Langton A : Filtration of microplastic spheres by biochar: removal efficiency and immobilisation mechanisms. *Water Res.* 2020;184:116165. 10.1016/j.watres.2020.116165 32688153

