# Supplementary material for: Micro(nano)plastics: invisible compounds with a visible impact
Source: F1000Res. 2024 Apr 19;13:69. Originally published 2024 Jan 15. [Version 2] doi: 10.12688/f1000research.142212.2 (PMC11040229; doi:10.12688/f1000research.142212.2)
Supplement: Peer review history

# Reviewer report: Reviewer response for version 2

**DOI:** 10.5256/f1000research.165058.r267915

**Published:** 2024-05-09

**Authors:** Hsuan-Cheng Lu

**Affiliations:** 1 Griffith University, Southport, Australia

The authors did not address some of my comments on the first version.

 After reading through the revised manuscript, I still think this review does not advance our knowledge and the content is rather simplistic compared to many published review articles. Although the authors stated that this is a mini review, there are several components missing when writing a good review on microplastics and nanoplastics.

 - Separation and Quantification Methods section is too simplistic (which is also acknowledged by another reviewer). 

 - Impact section is also lack of in-depth information. It is well known that abundance and characteristics (i.e., size, shape and polymer type) of microplastics and nanoplastics are critical factors of observed effects, which should have been included.

 - The authors attempted to present the lack of micro and nanoplastic studies conducted in groundwater, but there is not sufficient evidence to support the context based on the current level of details. The authors should have, for example, systematically reviewed the literature and included charts to show the number of studies in different aquatic matrices instead of simply performing narrative review.

Is the review written in accessible language?

Partly

Are all factual statements correct and adequately supported by citations?

Partly

Are the conclusions drawn appropriate in the context of the current research literature?

No

Is the topic of the review discussed comprehensively in the context of the current literature?

No

Reviewer Expertise:

microplastics; ecotoxicology

I confirm that I have read this submission and believe that I have an appropriate level of expertise to state that I do not consider it to be of an acceptable scientific standard, for reasons outlined above.

# Reviewer report: Reviewer response for version 1

**DOI:** 10.5256/f1000research.155732.r240000

**Published:** 2024-04-23

**Authors:** Prabhakar Singh

**Affiliations:** 1 Mahindra University, Hyderabad, India

Plastic-related research has gained prominence, especially in realizing the presence and spread of micro(nano)plastics (MNPs) in terrestrial and aquatic environments. However, there is limited focus on the fate and remediation of MNPs in soil and groundwater, and understanding the interaction of ubiquitous organic environmental pollutants with MNPs in biological systems is insufficient. This paper highlights the need to address MNP pollution by reducing plastic consumption, enhancing waste management practices, and developing environmentally friendly alternatives. It emphasizes the urgent need for policymakers and researchers to monitor and map MNP contamination in soil and groundwater for effective environmental protection.

 The review paper's strength lies in acknowledging the increasing significance of plastic-related research and highlighting the well-established presence of MNPs in terrestrial and aquatic environments. The authors advocate for future research to prioritize expanding the focus on plastic-related studies, specifically addressing the current limitations in understanding the fate and remediation of MNPs in soil and groundwater. They also emphasize the need for research efforts to uncover unknown aspects of the fate and bioaccumulation of ingested MNPs within the digestive tract of animals and to bridge the knowledge gap regarding the interaction of ubiquitous organic environmental pollutants with MNPs in biological systems.

Is the review written in accessible language?

Yes

Are all factual statements correct and adequately supported by citations?

Yes

Are the conclusions drawn appropriate in the context of the current research literature?

Yes

Is the topic of the review discussed comprehensively in the context of the current literature?

Yes

Reviewer Expertise:

Hydrology, watershed management, modeling, GIS application in drainage basin.

I confirm that I have read this submission and believe that I have an appropriate level of expertise to confirm that it is of an acceptable scientific standard.

# Reviewer report: Reviewer response for version 1

**DOI:** 10.5256/f1000research.155732.r251664

**Published:** 2024-04-09

**Authors:** Claude Kiki

**Affiliations:** 1 Institute of Urban Environment,, Chinese Academy of Sciences, Xiamen, China

The manuscript entitled “Micro(nano)plastics: invisible compounds with a visible impact” provides a comprehensive overview of the current understanding of micro(nano)plastics (NMPs), including their characterization methodologies, health impacts, sources, and potential remediation strategies. It is an informative piece that contributes significantly to the field. To further enhance the manuscript's clarity and depth, I recommend the following revisions for consideration.

1. In reference to Figure 1, it would be beneficial to delineate more explicitly the relationships among the depicted elements. An enhanced graphical representation could facilitate a clearer understanding of the connections and interactions between the various components related to NMPs. A revised figure that visually integrates these aspects with greater clarity would greatly benefit readers' comprehension.
2. The sentence beginning with "Plastics fragment into smaller..." and ending with "...to decompose in the environment" could benefit from a rephrasing to enhance clarity and precision.
3. The section addressing the identification and separation methodologies for NMPs would benefit from a more detailed exposition. It is recommended to structure this section into subsections, each dedicated to a specific method. Within each subsection, please provide a clear definition of the technique, its advantages, a summary of key findings from previous studies utilizing this method, and a discussion on its limitations. This structure will not only enrich the manuscript but also provide readers with a comprehensive understanding of the current methodological landscape.
4. The statement regarding "Synergistic effects of MNPs... after entering their bodies" requires rewriting for clarity and impact. A more precise and engaging sentence could better capture the nuances of MNPs' effects on biological systems.
5. The discussion on biodegradable and environmentally friendly remediation techniques for NMPs warrants further elaboration. It would be advantageous to detail specific methodologies, their mechanisms of action, efficacy, and potential environmental impacts. This expanded discussion could provide a critical evaluation of sustainable strategies for NMPs mitigation and offer valuable insights for future research directions.

Is the review written in accessible language?

Partly

Are all factual statements correct and adequately supported by citations?

Yes

Are the conclusions drawn appropriate in the context of the current research literature?

Partly

Is the topic of the review discussed comprehensively in the context of the current literature?

Partly

Reviewer Expertise:

Envuronmental Chemistry and Engineering

I confirm that I have read this submission and believe that I have an appropriate level of expertise to confirm that it is of an acceptable scientific standard, however I have significant reservations, as outlined above.

# Response

**Published:** 2024-04-14

**Authors:** Prabhakar Sharma

The manuscript entitled “Micro(nano)plastics: invisible compounds with a visible impact” provides a comprehensive overview of the current understanding of micro(nano)plastics (NMPs), including their characterization methodologies, health impacts, sources, and potential remediation strategies. It is an informative piece that contributes significantly to the field. To further enhance the manuscript's clarity and depth, I recommend the following revisions for consideration.

**Response: Many thanks for providing feedback to our paper.**

 In reference to Figure 1, it would be beneficial to delineate more explicitly the relationships among the depicted elements. An enhanced graphical representation could facilitate a clearer understanding of the connections and interactions between the various components related to NMPs. A revised figure that visually integrates these aspects with greater clarity would greatly benefit readers' comprehension.

**Response: I couldn’t improve the suggested figure (1) yet.**

 The sentence beginning with "Plastics fragment into smaller..." and ending with "...to decompose in the environment" could benefit from a rephrasing to enhance clarity and precision.

**Response: Thanks. The sentence has been rephrased.**

 The section addressing the identification and separation methodologies for NMPs would benefit from a more detailed exposition. It is recommended to structure this section into subsections, each dedicated to a specific method. Within each subsection, please provide a clear definition of the technique, its advantages, a summary of key findings from previous studies utilizing this method, and a discussion on its limitations. This structure will not only enrich the manuscript but also provide readers with a comprehensive understanding of the current methodological landscape.

**Response: Many thanks for this suggestion. As this is a mini review article, we couldn’t expand this section further.**

 The statement regarding "Synergistic effects of MNPs... after entering their bodies" requires rewriting for clarity and impact. A more precise and engaging sentence could better capture the nuances of MNPs' effects on biological systems.

**Response: This sentence has been modified.**

 The discussion on biodegradable and environmentally friendly remediation techniques for NMPs warrants further elaboration. It would be advantageous to detail specific methodologies, their mechanisms of action, efficacy, and potential environmental impacts. This expanded discussion could provide a critical evaluation of sustainable strategies for NMPs mitigation and offer valuable insights for future research directions.

**Response: This section is enhanced for more clarity.**

# Reviewer report: Reviewer response for version 1

**DOI:** 10.5256/f1000research.155732.r251662

**Published:** 2024-03-27

**Authors:** Hsuan-Cheng Lu

**Affiliations:** 1 Griffith University, Southport, Australia

The manuscript reviewed the research status of micro- and nanoplastics in soil and groundwater focusing on characterization methodologies, health impact, important source pathways, and available remediation options. However, the present manuscript does not advance our knowledge of any of the perspectives mentioned above because the level of details included is quite insufficient and does not meet the scientific standard for approval. There are many published review articles that elucidate the said topics in more detail. Hopefully the authors find the comments helpful.

 Upon reading, several major issues popped up:

- The authors attempted to present the lack of micro and nanoplastic studies conducted in groundwater, but there is not sufficient evidence to support the context based on the current level of details. The authors should have, for example, systematically reviewed the literature and included charts to show the number of studies in different aquatic matrices instead of simply performing narrative review.
- Again, the level of details in each section (method, impact, role and source pathway) is not enough and has already been widely presented by a lot of published reviews over the last few years (2400 reviews found on Scopus since 2020). The authors should try harder to expand these sections.
- Given the large number of publications, the authors should have cited review papers to support many statements throughout the manuscript instead of citing random case studies. For example, the authors only cited a case study for each technique in the first paragraph of separation and quantification methods.

Other minor comments:

- Title should be revised to better reflect the context of this review. For example, the authors should add information about soil and groundwater in the title.
- Figure 1 and Figure 2 fundamentally provide similar information, so I suggest the authors merging them similar to the graphical abstract in the study by Wu P, et al. 2019 (Ref 1)

Is the review written in accessible language?

Partly

Are all factual statements correct and adequately supported by citations?

Partly

Are the conclusions drawn appropriate in the context of the current research literature?

No

Is the topic of the review discussed comprehensively in the context of the current literature?

No

Reviewer Expertise:

microplastics; ecotoxicology

I confirm that I have read this submission and believe that I have an appropriate level of expertise to state that I do not consider it to be of an acceptable scientific standard, for reasons outlined above.

## References

[1] : Environmental occurrences, fate, and impacts of microplastics. *Ecotoxicol Environ Saf* .2019;184: 10.1016/j.ecoenv.2019.109612 109612 10.1016/j.ecoenv.2019.109612 31476450

# Response

**Published:** 2024-04-14

**Authors:** Prabhakar Sharma

Many thanks for your critical feedback. We have attempted to improve the revised version of the manuscript. As some of these comments are beyond the scope of this mini-review paper, we will work on all the suggested aspect in future comprehensive review paper.

# Reviewer report: Reviewer response for version 1

**DOI:** 10.5256/f1000research.155732.r239979

**Published:** 2024-03-18

**Authors:** Mansoor Ahmad

**Affiliations:** 1 Department of Environmental Engineering, Faculty of Engineering,, Eskişehir Technical University, Eskişehir, Turkey

- The title is interesting.
- The abstract can be more brief, and authors should highlight the article's aim.
- The micro (nano)plastics keyword can be removed as it is already in the title and replaced with another keyword.

Introduction

- Add reference to this line Plastic related research is increasingly in focus in recent times and more volume of studies are being pursued. [1],[2].
- Add reference to this line Plastics fragment into smaller pieces and get converted into MNPs through various processes.[3],[4].
- Add reference to this line  As a result,MNPs-related research has been getting significant attention in recent years, both in societies and in scientific communities, due to their potential impacts on ecological systems and human health. [5],[6].
- Add reference to this line  For example, the release of fiber from textiles, the presence of microbeads in cosmetic products, and the degradation of plastic debris could be potential sources of MNPs.[7]
- Add more information to the introduction about micro and nano plastics.

- I suggest the authors add more information about the sources, impacts and remediations of micro and nano plastics.

Is the review written in accessible language?

Yes

Are all factual statements correct and adequately supported by citations?

Partly

Are the conclusions drawn appropriate in the context of the current research literature?

Partly

Is the topic of the review discussed comprehensively in the context of the current literature?

Partly

Reviewer Expertise:

microplastics

I confirm that I have read this submission and believe that I have an appropriate level of expertise to confirm that it is of an acceptable scientific standard, however I have significant reservations, as outlined above.

## References

[1] : Scientometric Analysis of Microplastics across the Globe.2023; 10.1002/9781119879534.ch1 1-13 10.1002/9781119879534.ch1

[2] : Atmospheric micro (nano) plastics: future growing concerns for human health. *Air Qual Atmos Health* .2023;16(2) : 10.1007/s11869-022-01272-2 233-262 10.1007/s11869-022-01272-2 36276170 PMC9574822

[3] : Unveiling the overlooked threat: Macroplastic pollution in indoor markets in an urban city. *Case Studies in Chemical and Environmental Engineering* .2024;9: 10.1016/j.cscee.2023.100558 10.1016/j.cscee.2023.100558

[4] : A preliminary study on the natural aging behavior of microplastics in indoor and outdoor environments. *International Journal of Environmental Science and Technology* .2024;21(2) : 10.1007/s13762-023-05319-4 1923-1936 10.1007/s13762-023-05319-4

[5] : Mitigating microplastic pollution: A critical review on the effects, remediation, and utilization strategies of microplastics. *J Environ Manage* .2024;351: 10.1016/j.jenvman.2023.119988 119988 10.1016/j.jenvman.2023.119988 38181686

[6] : Microplastics in the environment: A critical overview on its fate, toxicity, implications, management, and bioremediation strategies. *Journal of Environmental Management* .2024;349: 10.1016/j.jenvman.2023.119433 10.1016/j.jenvman.2023.119433 39492398

[7] : Impact of Textile Product Emissions: Toxicological Considerations in Assessing Indoor Air Quality and Human Health. *springer* .2021; 10.1007/978-3-030-76073-1_27 10.1007/978-3-030-76073-1_27

# Response

**Published:** 2024-04-14

**Authors:** Prabhakar Sharma

The title is interesting.

**Response: We appreciate the reviewer for considering the importance of our paper.**

 The abstract can be more brief, and authors should highlight the article's aim.

**Response: We have attempted to revise the abstract by highlighting the paper objectives and reduced the length to some extent.**

 The micro (nano)plastics keyword can be removed as it is already in the title and replaced with another keyword.

**Response: Thanks. We have removed the micro(nano)plastics keyword from the paper.**

 Introduction

 Add reference to this line Plastic related research is increasingly in focus in recent times and more volume of studies are being pursued. [1],[2].

**Response: Both references are added as suggested by the reviewer.**

 Add reference to this line Plastics fragment into smaller pieces and get converted into MNPs through various processes.[3],[4].

**Response: Both references are added as suggested by the reviewer.**

 Add reference to this line  As a result,MNPs-related research has been getting significant attention in recent years, both in societies and in scientific communities, due to their potential impacts on ecological systems and human health. [5],[6].

**Response: Both references are added as suggested by the reviewer.**

 Add reference to this line  For example, the release of fiber from textiles, the presence of microbeads in cosmetic products, and the degradation of plastic debris could be potential sources of MNPs.[7]

**Response: The suggested reference has been included.**

 Add more information to the introduction about micro and nano plastics.

**Response: Many thanks. We have attempted to expand this aspect and included more discussion about MNPs in the Introduction section.**

 I suggest the authors add more information about the sources, impacts and remediations of micro and nano plastics.

**Response: We have included more discussion about sources and impact in “Source Pathways of MNPs” section and remediation part in “Innovative Solutions and Ongoing Efforts for Mitigation of MNPs” section.**

 References

 2. Bhat M, Eraslan F, Gaga E, Gedik K: Scientometric Analysis of Microplastics across the Globe. 2023. 1-13 Publisher Full Text

 3. Bhat MA, Gedik K, Gaga EO: Atmospheric micro (nano) plastics: future growing concerns for human health.Air Qual Atmos Health. 2023; 16 (2): 233-262 PubMed Abstract | Publisher Full Text

 5. Bhat M: Unveiling the overlooked threat: Macroplastic pollution in indoor markets in an urban city. Case Studies in Chemical and Environmental Engineering. 2024; 9. Publisher Full Text

 6. Bhat M, Gedik K, Gaga E: A preliminary study on the natural aging behavior of microplastics in indoor and outdoor environments. International Journal of Environmental Science and Technology. 2024; 21 (2): 1923-1936 Publisher Full Text

 8. Thacharodi A, Hassan S, Meenatchi R, Bhat MA, et al.: Mitigating microplastic pollution: A critical review on the effects, remediation, and utilization strategies of microplastics.J Environ Manage. 2024; 351: 119988 PubMed Abstract | Publisher Full Text

 9. Thacharodi A, Meenatchi R, Hassan S, Hussain N, et al.: Microplastics in the environment: A critical overview on its fate, toxicity, implications, management, and bioremediation strategies. Journal of Environmental Management. 2024; 349. Publisher Full Text

 31. Bhat, M Eraslan, F Gedik, K Gaga, et al.: Impact of Textile Product Emissions: Toxicological Considerations in Assessing Indoor Air Quality and Human Health. springer. 2021. Publisher Full Text
